# Laboratory Data Timeliness and Completeness Improves Following Implementation of an Electronic Laboratory Information System in Côte d’Ivoire: Quasi-Experimental Study on 21 Clinical Laboratories From 2014 to 2020

**DOI:** 10.2196/50407

**Published:** 2024-03-20

**Authors:** Yao He, Yves-Rolland Kouabenan, Paul Henri Assoa, Nancy Puttkammer, Bradley H Wagenaar, Hong Xiao, Stephen Gloyd, Noah G Hoffman, Pascal Komena, N'zi Pierre Fourier Kamelan, Casey Iiams-Hauser, Adama Sanogo Pongathie, Alain Kouakou, Jan Flowers, Nadine Abiola, Natacha Kohemun, Jean-Bernard Amani, Christiane Adje-Toure, Lucy A Perrone

**Affiliations:** 1 Digital Initiatives Group at International Training and Education Center for Health, Department of Global Health Schools of Public Health and Medicine University of Washington Seattle, WA United States; 2 International Training and Education Center for Health - Côte d'Ivoire Abidjan Cote D'Ivoire; 3 Department of Global Health Schools of Public Health and Medicine University of Washington Seattle, WA United States; 4 Department of Epidemiology, Schools of Public Health and Medicine University of Washington Seattle, WA United States; 5 Public Health Sciences Division Fred Hutchinson Cancer Center Seattle, WA United States; 6 Department of Pathology and Laboratory Medicine University of Washington Seattle, WA United States; 7 Direction de l’Informatique et de l’Information Sanitaire Ministry of Health, Public Hygiene and Universal Health Coverage Abidjan Cote D'Ivoire; 8 Laboratory Branch United States Centers for Disease Control and Prevention Abidjan Cote D'Ivoire; 9 Retro-CI Laboratory United States Centers for Disease Control and Prevention Abidjan Cote D'Ivoire; 10 Department of Pathology and Laboratory Medicine University of British Columbia (UBC) Vancouver, BC Canada

**Keywords:** clinical laboratory, electronic laboratory information system, data quality, evaluation, impact evaluation, time series, laboratory, information system, information systems, HIV, sexually transmitted infection, sexually transmitted disease, sexually transmitted, lab, labs, adoption, implementation, effectiveness

## Abstract

**Background:**

The Ministry of Health in Côte d'Ivoire and the International Training and Education Center for Health at the University of Washington, funded by the United States President’s Emergency Plan for AIDS Relief, have been collaborating to develop and implement the Open-Source Enterprise-Level Laboratory Information System (OpenELIS). The system is designed to improve HIV-related laboratory data management and strengthen quality management and capacity at clinical laboratories across the nation.

**Objective:**

This evaluation aimed to quantify the effects of implementing OpenELIS on data quality for laboratory tests related to HIV care and treatment.

**Methods:**

This evaluation used a quasi-experimental design to perform an interrupted time-series analysis to estimate the changes in the level and slope of 3 data quality indicators (timeliness, completeness, and validity) after OpenELIS implementation. We collected paper and electronic records on clusters of differentiation 4 (CD4) testing for 48 weeks before OpenELIS adoption until 72 weeks after. Data collection took place at 21 laboratories in 13 health regions that started using OpenELIS between 2014 and 2020. We analyzed the data at the laboratory level. We estimated odds ratios (ORs) by comparing the observed outcomes with modeled counterfactual ones when the laboratories did not adopt OpenELIS.

**Results:**

There was an immediate 5-fold increase in timeliness (OR 5.27, 95% CI 4.33-6.41; *P*<.001) and an immediate 3.6-fold increase in completeness (OR 3.59, 95% CI 2.40-5.37; *P*<.001). These immediate improvements were observed starting after OpenELIS installation and then maintained until 72 weeks after OpenELIS adoption. The weekly improvement in the postimplementation trend of completeness was significant (OR 1.03, 95% CI 1.02-1.05; *P*<.001). The improvement in validity was not statistically significant (OR 1.34, 95% CI 0.69-2.60; *P*=.38), but validity did not fall below pre-OpenELIS levels.

**Conclusions:**

These results demonstrate the value of electronic laboratory information systems in improving laboratory data quality and supporting evidence-based decision-making in health care. These findings highlight the importance of OpenELIS in Côte d'Ivoire and the potential for adoption in other low- and middle-income countries with similar health systems.

## Introduction

Clinical laboratories provide critically important data for disease investigations and supply timely information for public health. Laboratory results help identify and characterize pathogens, conduct routine surveillance, and respond to communicable and noncommunicable diseases. Effective and timely laboratory services help achieve national disease priorities and global targets including the Sustainable Development Goals and the 95-95-95 targets launched by the Joint United Nations Programme on HIV/AIDS [1-5]. Clusters of differentiation 4 (CD4) testing is critical for monitoring the immune status of people living with HIV and the risk of opportunistic infections. Health care providers depend on laboratory results to decide on differentiated care models and tuberculosis screening [6]. For a national HIV program to be highly successful and reduce HIV-related deaths, the health system must integrate data from an effective clinical laboratory system. Access to quality laboratory services remains a challenge in low- and middle-income countries (LMICs), and many still rely on paper-based records [7]. Throughout the cascade of care, paper documents and records require a careful chain of possession, tracking, and version control, and their use and sometimes loss can impede an already complex process of service delivery. Problems in data quality could occur at every step of this manual process, jeopardizing the quality of care.

Laboratory information systems (LISs) are an important part of the infrastructure for laboratory operation and information management. According to the requirements for quality and competence of medical laboratories issued by the International Organization for Standardization (ISO), most of the information management requirements are for electronic systems [8]. This signals that, to reach ISO standards, laboratories should use electronic rather than paper-based LISs. The World Health Organization established the Stepwise Laboratory Quality Improvement Process Toward Accreditation (SLIPTA) checklist to guide clinical laboratories in Africa through continuous quality improvement toward accreditation to international standards [9]. The SLIPTA checklist highlights computerized LISs as an integral step to ensuring laboratory operation and fulfilling the criteria of laboratory information management on ensuring data quality, data storage and backup, and patient confidentiality [9]. The lack of effective electronic LISs is one of the barriers to quality laboratory service delivery in LMICs [10].

LISs can also improve the quality of care, patient safety, and disease surveillance [10-14]. Compared to paper-based information systems, electronic LISs provide more timely and more accurate monitoring and reporting of turnaround time, test failure rates, and other laboratory quality indicators essential to informing clinical care [10]. The features in the Open-Source Enterprise-Level Laboratory Information System (OpenELIS) are intended to ensure data timeliness, completeness, and validity. Control elements and graphical widgets such as drop-down menus and radio buttons make data entry easier and faster, supporting data timeliness and validity. Required data fields must be completed to generate the laboratory result for a patient, assuring data completeness. Valid ranges displayed alongside each test result, the flags for manual review of out-of-range results, and the mandatory validation step before generating results help ensure data validity.

The International Training and Education Center for Health (I-TECH) has been collaborating with the Ministry of Health, Public Hygiene, and Universal Health Coverage (MSHPCMU) in Côte d'Ivoire since 2009 with funding from the United States President’s Emergency Plan for AIDS Relief to build a functional, accredited diagnostic and laboratory network for effective HIV care and treatment. One of the key program interventions is establishing and supporting the use of OpenELIS interfacing with laboratory testing analyzers [15]. From 2009 to 2021, OpenELIS was installed at 108 laboratories in Côte d'Ivoire, including 68 sites that use it primarily for HIV data, 1 for food and drug safety data, 27 for tuberculosis data only, and 12 sites that use OpenELIS for routine laboratory testing data.

This study aims to estimate the effect of OpenELIS on data quality indicators including data completeness, timeliness, and validity, using CD4 testing data. Specifically, we aimed to answer the following questions: (1) Did the use of OpenELIS in Côte d’Ivoire’s clinical laboratories lead to statistically significant improvements in the data quality outcomes? (2) If so, what was the magnitude of the improvements compared to the counterfactual scenario in which the laboratories had not adopted OpenELIS? and (3) Had the improvements persisted after the initial adoption of OpenELIS?

Beyond a single study of the effect of an LIS on laboratory turnaround time in Peru [16], there is limited evidence on how LIS interventions enhance data quality in LMICs. Findings from our study will provide evidence on the effectiveness of the OpenELIS intervention in improving data quality; inform policy recommendations and guidance for the Côte d'Ivoire MSHPCMU; and inform decision makers elsewhere to consider adopting OpenELIS in countries with a context similar to Côte d'Ivoire.

## Methods

### Study Design and Data Sources

**Figure 1:**
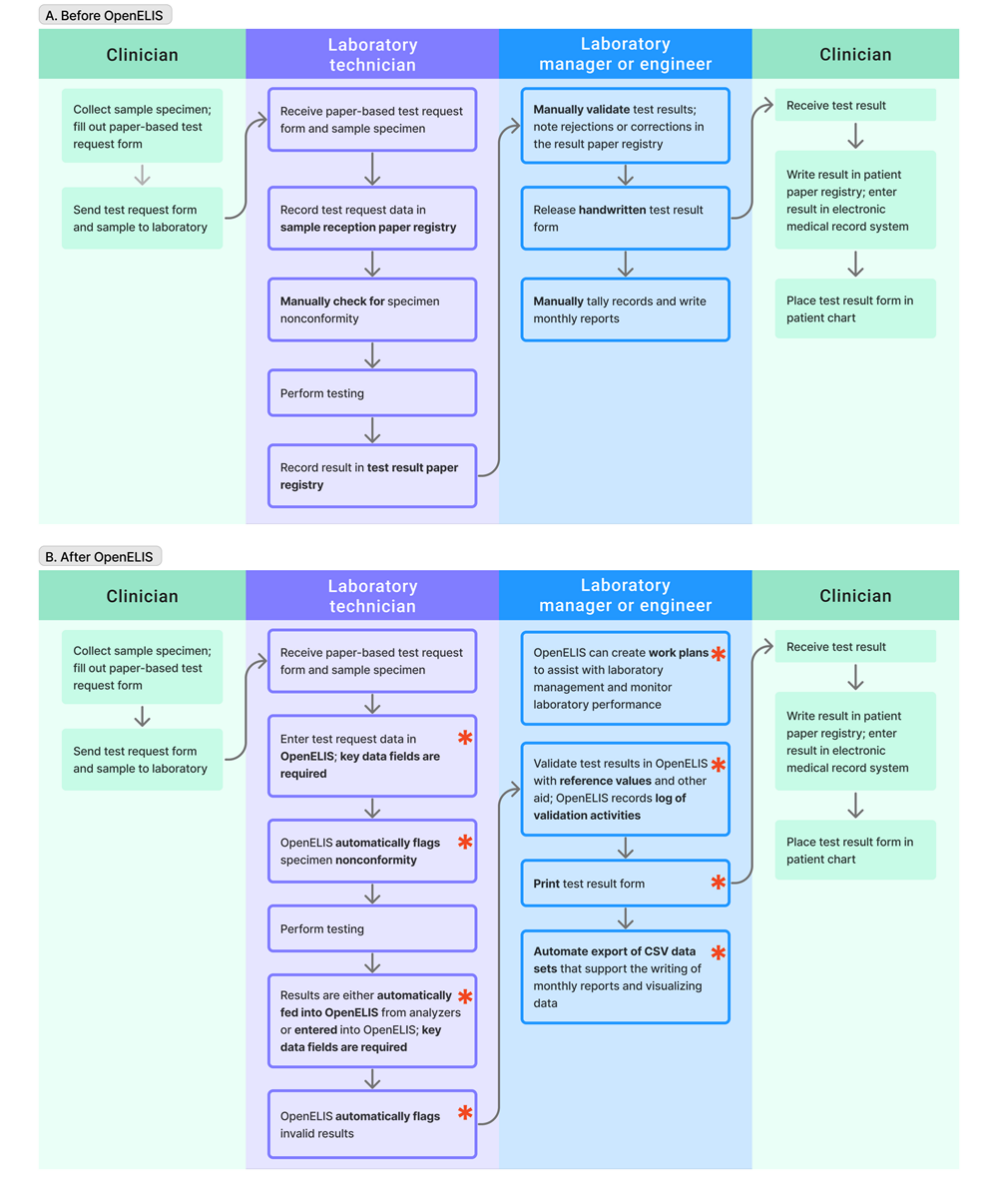
Data flow process for a typical clinical laboratory test in Côte d'Ivoire (A) before and (B) after adopting OpenELIS. * denotes the difference between OpenELIS and a paper-based laboratory information system. CSV: comma-separated values; OpenELIS: Open-Source Enterprise-Level Laboratory Information System.

We conducted an interrupted time-series analysis using weekly time-series data. The interrupted time-series analysis is a quasi-experimental design in the evaluation of health care interventions or policies that are introduced at a specific time [17,18]. We hypothesized that, compared to paper registries, OpenELIS would improve laboratory data quality measured by 3 primary outcomes, that is, timeliness, completeness, and validity (Figure 1). Timeliness was defined as the proportion of test results produced within 1 day upon receiving test samples. Completeness was defined as the proportion of test results having complete data on all 4 required data fields, namely patient identifier, age, sex, and result date. Validity was defined as the proportion of CD4 cell count test results that were within the valid range (0-2000 cells/mm^3^).

**Figure 2:**
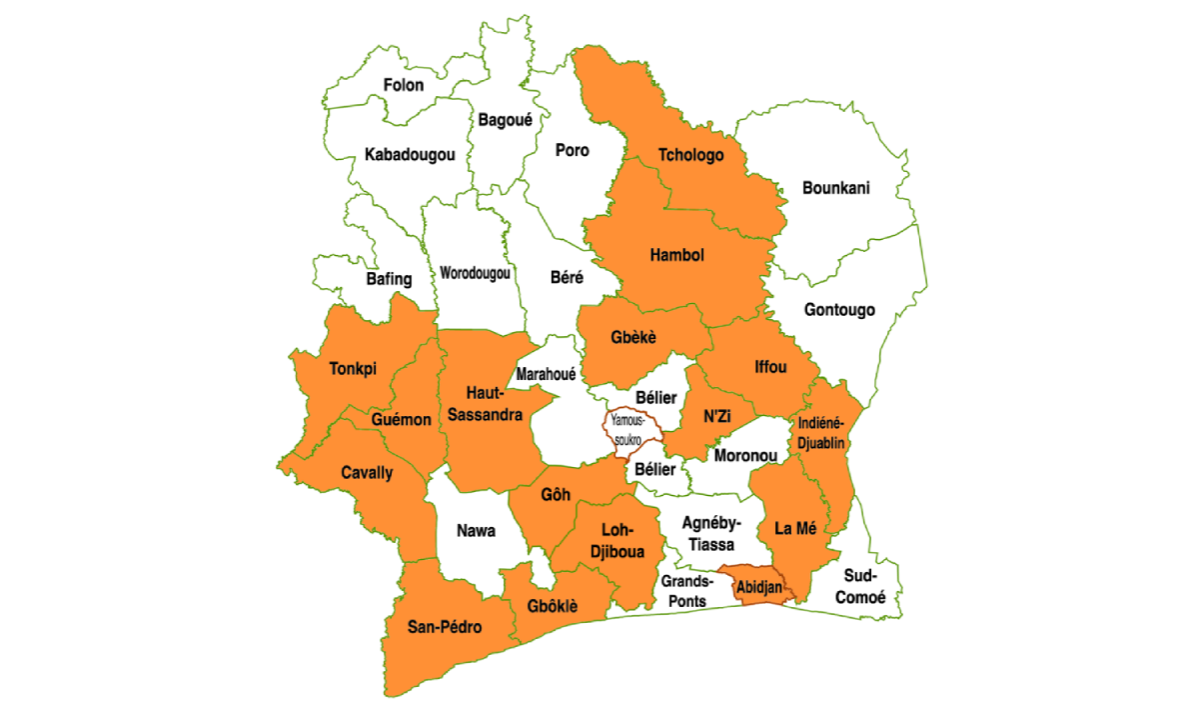
The 13 health regions in Côte d’Ivoire where data were collected from paper registries and OpenELIS for the evaluation of the effect of OpenELIS on data quality in Côte d’Ivoire from 2014-2020. OpenELIS: Open-Source Enterprise-Level Laboratory Information System. Adapted from Wikimedia [19], which is published under Creative Commons Attribution 4.0 International License [20].

We collected time-series data in January 2021 from 21 clinical laboratories across 13 health regions in Côte d'Ivoire that started using OpenELIS between 2014 and 2020 (Figure 2). The time frame of the time-series data covered up to 48 weeks before a laboratory started using OpenELIS until up to 72 weeks after. This sampling considered adequate representation in terms of geography and service capacity, as well as the feasibility of data collection. A total of 5 sites were regional reference laboratories that provided HIV viral load testing services, 3 were regional laboratories that were not reference sites for HIV viral load testing, and 13 were part of either at general hospitals or urban hospitals that provided HIV-related laboratory services at the district level. We abstracted deidentified, individual-level patient data for all outcomes from laboratory paper registries for the preimplementation period and from local OpenELIS servers for the postimplementation period. Data were aggregated by laboratory and organized by week to model the outcomes. The number of laboratories whose data were incorporated in the analyses for timeliness, completeness, and validity was 8, 21, and 20, respectively. The reasons why the number of laboratories for each outcome was different are the following. To estimate the timeliness of the data in paper registries, we needed to link individual-level records from test sample reception registries with those from test result registries by unique medical record number. A total of 13 of the 21 sampled laboratories did not have any sample reception registries from before they started using OpenELIS, so we could only estimate changes in timeliness at 8 laboratories. Accidents such as fire and flood were the most common reasons why laboratories were missing paper records, and some laboratories destroyed paper records older than 5 years because the national policy only requires 5 years for archiving paper records. To estimate completeness, we had all necessary data from all 21 sampled sites. To estimate validity, we needed data on CD4 cell counts, but 1 laboratory did not record CD4 cell counts in OpenELIS. Therefore, we could only estimate changes in validity for 20 sites.

### Statistical Analysis

We used a mixed-effects, segmented logistic regression model to examine the trend of the primary outcomes and their changes associated with OpenELIS implementation. We estimated odds ratios (ORs) representing weekly outcomes compared with outcomes that would have occurred if the laboratories had not adopted OpenELIS. We reported both the level change and slope change, specifically investigating the abrupt changes in outcomes observed during the first week of OpenELIS implementation and the weekly changes in the trend starting from the second week of OpenELIS implementation.

We included fixed-effect monthly indicator variables in the models to account for seasonal effects, random intercepts to account for clustering at the laboratory level, and random slopes over time. We also accounted for the weekly number of HIV clients recorded at a given laboratory as weight. The logistic regression model equation we used was:



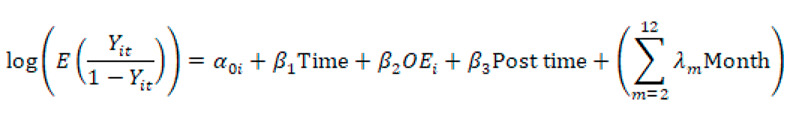



where *i* indicates laboratory and *t* indicates week as the time unit. *Y_it_* represents the proportion of laboratory records achieving each of the 3 primary outcomes comparing the factual to the counterfactual; *α*_0_*_i_* represents the model intercept with a fixed effect and a random effect at the laboratory level; *Time* counts the weeks from the earliest preimplementation time point until the latest postimplementation time point; *OE_i_* is the dummy variable indicating the weeks when a laboratory *i* was using OpenELIS (1 for the first week when a laboratory started using and thereafter and 0 otherwise); *Post time* counts the weeks after the first week of implementation; and *Month* is an individual dummy variable indicating the month of the year with January as the reference.

To develop expected counterfactual forecasts, we reran the logistic models to extend preimplementation trends through 72 weeks after a laboratory started using OpenELIS as a counterfactual to the observed postimplementation trends. The mixed-effects models accounted for data that were missing at certain weeks using standard maximum likelihood estimation. To visualize the factual and counterfactual trends across time, we plotted the predicted proportions of test results achieving the primary outcomes under the 2 scenarios and the 95% prediction intervals for the factual estimates accounting for random variations at the laboratory level. All analyses were conducted using R (version 4.1.0; R Core Team) and a 2-sided α value of .05.

### Ethical Considerations

This study was determined to be nonhuman subject research by the University of Washington institutional review board and the United States Centers for Disease Control and Prevention (CDC). The study was approved by Côte d'Ivoire Comité National d'Ethique des Sciences de la Vie et de la Santé (CNESVS, Ivorian Institutional Review Board; reference 006-21/MSHP/CNESVS-km). The US CDC reviewed this research and paper but had no influential role in the study design, data collection, analysis, and interpretation.

## Results

The number of laboratory weeks in the analyses were 613, 1820, and 1629 for timeliness, completeness, and validity, respectively. The analysis included an average of 34 weeks of preimplementation data and 53 weeks of postimplementation data from each of the 21 laboratories. A total of 24,381 and 40,040 HIV client records from the pre- and postimplementation periods, respectively, were in the analysis (Table 1).

Only 3 of the 21 laboratories had all 48 weeks of complete data on sample reception before the laboratories adopted OpenELIS and when they used paper registries exclusively. A total of 10 laboratories had all 48 weeks of complete data on test results. After adopting OpenELIS, 19 of the 21 laboratories had 72 weeks of complete data for both sample reception and test results.

**Figure 3:**
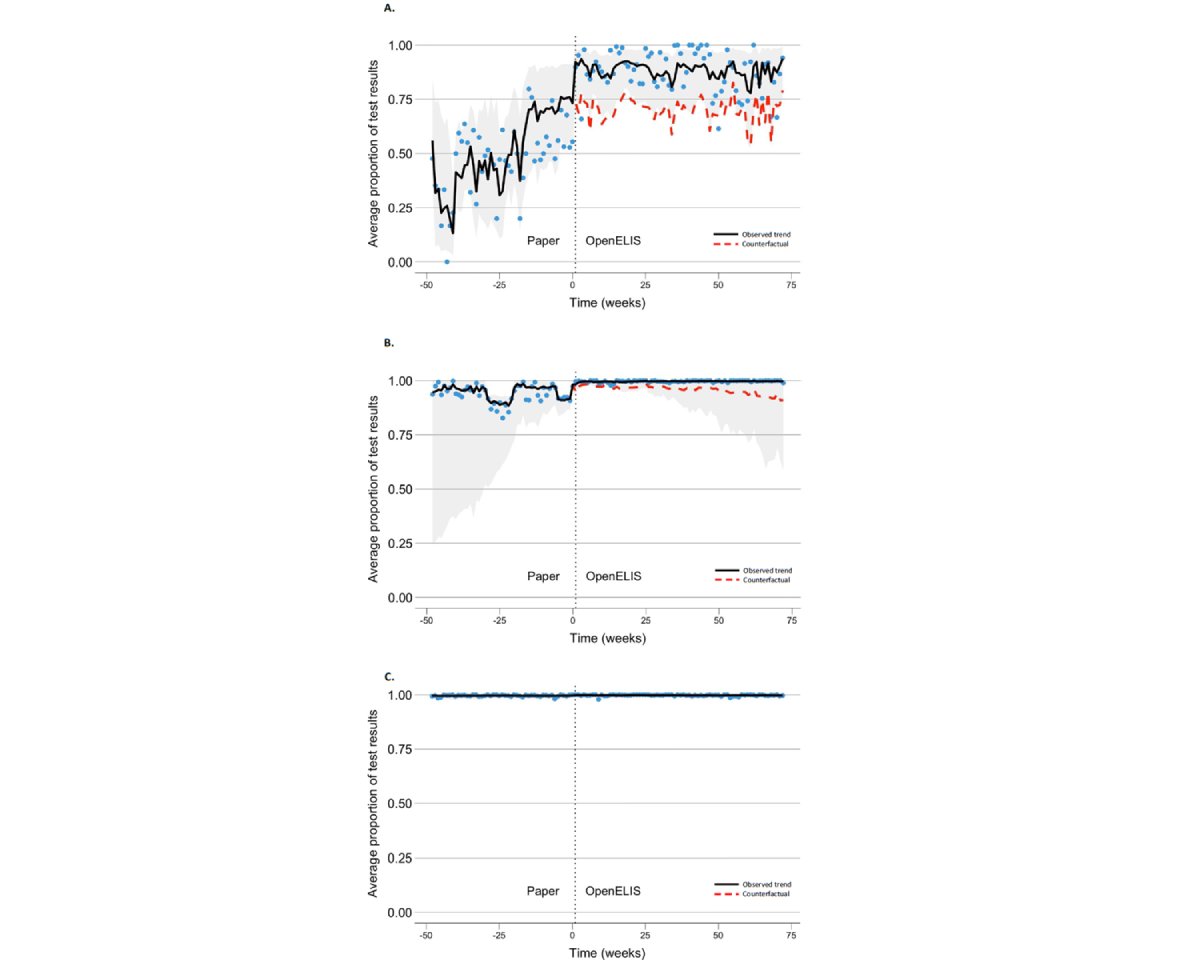
Laboratory test result timeliness, completeness, and validity from 48 weeks before OpenELIS implementation to 72 weeks after at clinical laboratories in Côte d’Ivoire. (A) Timeliness: average proportion of test results reported within 1 day of test sample reception; (B) Completeness: average proportion of test results having complete information for all required data fields; (C) Validity: average proportion of test results having valid results. Vertical dotted line: first week when a laboratory started using OpenELIS. Blue dots: weekly grand means of the observed outcomes at all laboratories. Black solid line: estimated trend around the observed data points. Light gray area: 95% prediction interval around the estimated observed trend. Red dashed line: counterfactual estimates if there were no OpenELIS. OpenELIS: Open-Source Enterprise-Level Laboratory Information System.

Before adopting OpenELIS, a typical laboratory had minor improvements in the 3 data quality outcomes that were not statistically significant (timeliness: OR 1.03, 95% CI 0.99-1.07; *P*=.12; completeness: OR 1.11, 95% CI 0.93-1.32; *P*=.24; and validity: OR 1.00, 95% CI 0.99-1.02; *P*=.76; Table 2). Within the first week of OpenELIS implementation, we observed an immediate level change in data timeliness, completeness, and validity (Figure 3). After adopting OpenELIS, the laboratories had 5.27-fold greater odds of timely results (OR 5.27, 95% CI 4.33-6.41; *P*<.001); 3.59-fold greater odds of complete data for the required fields (OR 3.59, 95% CI 2.40-5.37; *P*<.001); and 1.34-fold greater odds of valid results (OR 1.34, 95% CI 0.69-2.60; *P*=.38; Table 2). The immediate improvements in timeliness and completeness were both programmatically substantial and statistically significant.

Starting from the second week of OpenELIS implementation, we observed a slope change in the trend of completeness. The average odds of test results having complete data for the required fields each week thereafter were 1.03 times the odds had the laboratories not started using OpenELIS (OR 1.03, 95% CI 1.02-1.05; *P*<.001). In fact, the average proportion of test results having complete data stayed at almost 100% after a laboratory started using OpenELIS, while the average proportion declined over time in the counterfactual had there been no OpenELIS (Figure 3B). There was no significant change in the trend of timeliness and validity (timeliness: OR 1.01, 95% CI 0.99-1.02; *P*=.49 and validity: OR 0.99, 95% CI 0.97-1.01; *P*=.55).

In terms of heterogeneity at the laboratory level, laboratories at local general hospitals seemed to have more substantial improvements in timeliness immediately after starting to use OpenELIS and have maintained the improvements better over time as compared to regional laboratories (Figure S1 in Multimedia Appendix 1). Since a greater number of regional laboratories had less complete data than laboratories at general hospitals before OpenELIS implementation, immediate improvements in completeness were more obvious at regional laboratories, and the improvements also maintained over time (Figure S2 in Multimedia Appendix 1). Consistent with the result at the aggregate level, no laboratories had changes in validity (Figure S3 in Multimedia Appendix 1).

**Table 1:** Background information on the clinical laboratories in Côte d’Ivoire included in this study, before and after the adoption of OpenELIS^a,b^. All laboratories had data that could be included in the analyses on completeness and validity unless otherwise noted.

Health region	Clinical laboratories, n	Weeks of data collected, n		HIV client records, n	
		Before	After	Before	After
All regions	21	34	53	24,381	40,040
Abidjan II	3	35	68	4780	10,453
Agneby-Tiassa-Me	1	48	61	2022	1756
Cavally-Guemon	3	45	39	2718	1177
Gbeke	1	8	70	306	3505
Gbokle-Nawa-San Pedro	3	27	65	2769	8383
Goh	1	39	23	538	218
Hambol^c^	1	14	31	401	303
Haut Sassandra	1	32	69	1105	582
Indenie-Djuablin	2	32	51	4015	6857
Loh Djiboua	1	35	64	1164	1741
N'zi-Iffou	2	46	30	2995	888
Poro-Tchologo-Bagoue	1	44	42	719	503
Tonkpi	1	14	69	849	3674

^a^The time period for this evaluation was from 2014-2020.

^b^OpenELIS: Open-Source Enterprise-Level Laboratory Information System.

^c^The laboratory sampled in this region did not have data that could be included in the analysis on validity.

**Table 2:** Estimated ratios comparing odds of achieving the quality outcomes of timeliness, completeness, or validity using specifically CD4^a^ testing data, each week to the counterfactual scenario where the clinical laboratories in Côte d’Ivoire had not adopted OpenELIS^b,c^.

	Preadoption weekly change in the slope of the outcome		Immediate level change during first week of OpenELIS implementation		Postadoption weekly change in slope of the outcome	
	OR^d^ (95% CI)	*P* value	OR (95% CI)	*P* value	OR (95% CI)	*P* value
Data timeliness	1.03 (0.99-1.07)	.12	5.267 (4.33-6.41)	<.001	1.005 (0.99-1.02)	.49
Data completeness	1.11 (0.93-1.32)	.24	3.594 (2.40-5.37)	<.001	1.034 (1.02-1.05)	<.001
Data validity	1.00 (0.99-1.02)	.76	1.341 (0.69-2.60)	.38	0.994 (0.97-1.01)	.55

^a^CD4: clusters of differentiation 4.

^b^The time period for this evaluation was 2014-2020.

^c^OpenELIS: Open-Source Enterprise-Level Laboratory Information System.

^d^OR: odds ratio.

## Discussion

This study shows that data availability improved after clinical laboratories in Côte d’Ivoire adopted OpenELIS, and there was rapid and substantial improvement in data timeliness and completeness. Following the immediate post-OpenELIS implementation improvements, both timeliness and completeness remained close to 100%. Data validity was very close to 100% both before and after OpenELIS adoption. There was some heterogeneity in the results at the laboratory level, with laboratories at local general hospitals showing more substantial improvements in timeliness while regional laboratories showed more obvious immediate improvements in completeness.

To our knowledge, this is the first study to demonstrate the immediate and maintained effect of a scaled, multisite LIS on data quality in sub-Saharan Africa. There are limited studies from LMICs despite significant financial investments from the international donor community. Previous studies from LMICs took place on other continents or did not include as many laboratories or subnational geographic areas as ours did. The e-Chasqui LIS for tuberculosis testing data at 14 laboratories in Peru was the only one that was implemented and evaluated through a cluster randomized controlled trial [21]. The trial showed that the LIS decreased turnaround time and improved quality of care, but the study did not examine the effect on data quality [21]. Our study contributes to the evidence by quantifying the effect size of data quality improvement and its change over a period of 30 months at 21 clinical laboratories. The findings offer critical information for policy makers and digital health researchers in LMICs when considering the real-world effectiveness of the OpenELIS software or digitalization of a sub-Saharan African laboratory system.

Systematic implementation of a single LIS across an entire national health system is rare in LMICs. A rapid literature review of peer-reviewed journal articles identified 8 different LISs that 6 countries have implemented at more than 1 site nationally, namely Ethiopia, Malawi, Namibia, South Africa, Peru, and Vietnam [16,21-30]. The LIS in South Africa is the only 1 that has the capacity to record data on all laboratory testing services at all clinical laboratories in the country [22]. It is likely that, as a result of the nationwide LIS adoption in South Africa, usage of laboratory data in research has also increased, since almost 20% of the rapid literature review search results from LMICs were epidemiological studies from South Africa using data from the LIS [31-39].

Part of the success of OpenELIS in Côte d’Ivoire can be attributed to the systematic approach taken by I-TECH in establishing and supporting the use of OpenELIS. The approach included the endorsement by the MSHPCMU and locally trained technical staff who facilitated implementation and provided continuous technical support of the system to the laboratories and their users. The relative advantages of OpenELIS compared to paper registries, ease of implementation, and continuous technical support have facilitated the routine use of OpenELIS. In addition, an experienced software development team designed various functional and interface features of the software specifically intended to improve data quality, for example, drop-down menus for fast and accurate selections and automatic data completeness and validity checks. The decade-long development and implementation of OpenELIS in Côte d’Ivoire has been open-source, iterative, and collaborative so that feedback from users, laboratory managers, and governing bodies was directly incorporated into the software to match the needs of the users [15].

Our study provides evidence for improving laboratory data quality, which is important for the improvement of clinical care and treatment. A study from Kenya identified that reduced turnaround time and timely communication of laboratory test results are some of the key factors for improving HIV treatment retention [12].

In addition, in terms of patient safety, LISs reduced the probability of errors in patient identification and subsequent inappropriate treatment by reducing manual clerical work through automation and user-friendly widgets [10]. A survey of clinicians in Malawi revealed that laboratories’ poor documentation of test results was one of the reasons for having little trust in laboratory capability and not frequently using laboratory test results in patient management [13]. Last but not least, LISs can also improve disease surveillance by making it easier to record accurate demographic data that inform disease classification, assessment of population-specific rates, and contact tracing for infectious diseases [11], as well as to report laboratory-confirmed diagnoses to surveillance systems that allow more accurate estimation of disease burdens [14].

This evaluation has many strengths and some limitations. The use of multiple data points before and after OpenELIS helps capture the changes in time-varying variables through the data we used to generate the pre- and posttrends. However, since all sites that have been using OpenELIS, including the ones sampled in this evaluation, were purposefully selected due to their higher capacity, they might also have had a higher probability of adhering to the OpenELIS user instructions. The regression models did not specifically account for potential confounding effects from the COVID-19 pandemic and related control measures that were enforced from March to July 2020 mostly in the Greater Abidjan area in Côte d’Ivoire [40]. However, since the percentage of weekly data points occurring after March 2020 was low (3.3% for timeliness, 7.0% for completeness, and 7.7% for validity), and most of the data points came outside Abidjan, we thought that the influence of the pandemic on our outcomes was low. Furthermore, the 3 outcomes were proportions of the total testing data, so the estimates were less likely to be influenced by fluctuations in total testing volume due to the pandemic. Nonetheless, we still included the total number of tests performed by a laboratory as weight in the regression model. The results may be generalizable to remaining clinical laboratories in Côte d’Ivoire that are at the same tiers in the health system as the sites sampled in this study, but generalizability to laboratories in other LMICs needs further studies to demonstrate.

The implementation of an LIS can have a significant effect on improving data quality in LMICs. This study found that the implementation of OpenELIS led to improved timeliness and completeness of laboratory data. The system also facilitated better data availability compared to paper registries, enabling health care providers to make informed decisions based on accurate and up-to-date information.

The effectiveness of an LIS depends on many factors beyond the capabilities of the LIS itself, including systematic planning for adoption, supportive technical and implementation staff, attitudes of policy makers and users, presence of supportive policies, financial resources, infrastructure, and organizational and individual readiness to adopt innovations. Future research is needed to explore the facilitators and barriers to implementing health information systems such as an LIS in LMICs.

## Appendix

Multimedia Appendix 1 Laboratory test result data timeliness, completeness, and validity at individual laboratories from 48 weeks before OpenELIS implementation to 72 weeks after at clinical laboratories in Côte d’Ivoire.

## Abbreviations

CDC: Centers for Disease Control and Prevention
CD4: clusters of differentiation 4
CNESVS: Côte d'Ivoire Comité National d'Ethique des Sciences de la Vie et de la Santé
ISO: International Organization for Standardization
I-TECH: International Training and Education Center for Health
LIS: laboratory information system
LMIC: low- and middle-income country
MSHPCMU: Ministry of Health, Public Hygiene, and Universal Health Coverage
OpenELIS: Open-Source Enterprise-Level Laboratory Information System
OR: odds ratio
SLIPTA: Stepwise Laboratory Quality Improvement Process Toward Accreditation

## Data Availability

The data sets generated and analyzed during this study are available from the corresponding author on reasonable request.
