# Supplementary material for: Laboratory Data Timeliness and Completeness Improves Following Implementation of an Electronic Laboratory Information System in Côte d’Ivoire: Quasi-Experimental Study on 21 Clinical Laboratories From 2014 to 2020
Source: JMIR Public Health Surveill. 2024 Mar 20;10:e50407. doi: 10.2196/50407 (PMC10993113; doi:10.2196/50407)
Supplement: Multimedia Appendix 1 [file publichealth_v10i1e50407_app1.docx]

**Figure S1.** Timeliness—average proportion of test results reported within 1 day of test sample reception.


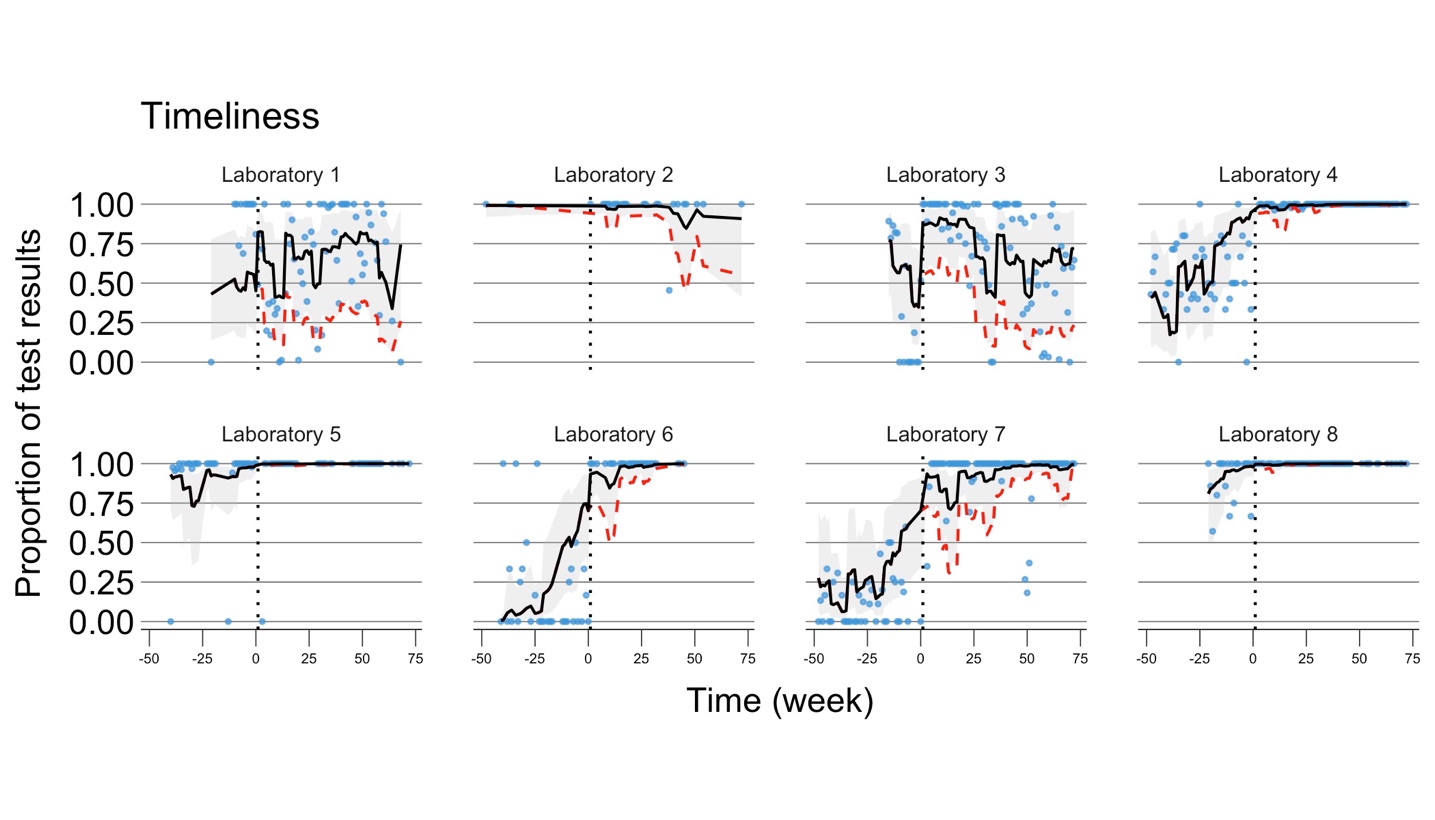


**Figure S2.** Completeness—average proportion of test results having complete information for all required data fields.


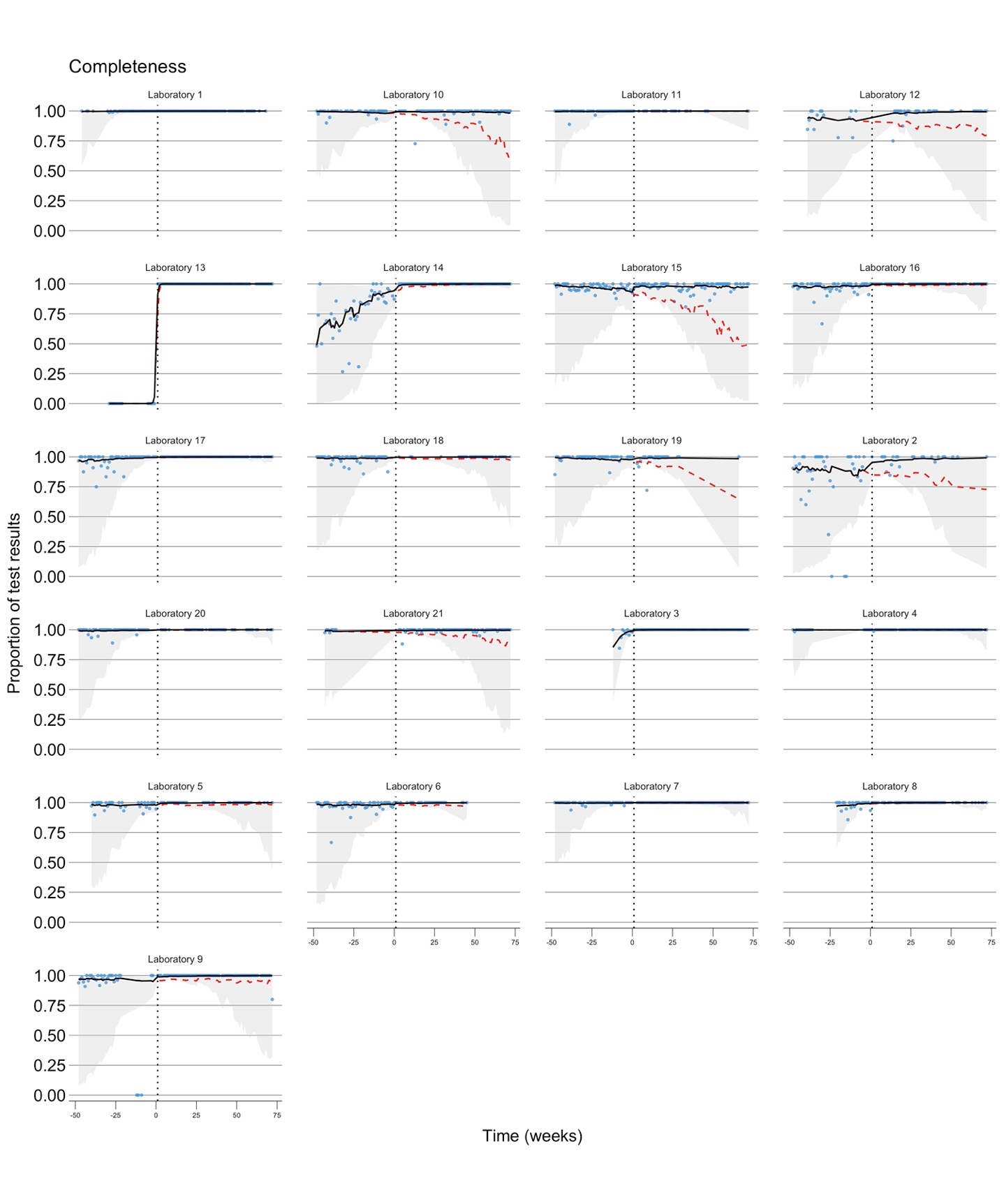


**Figure S3.** Validity—average proportion of test results having valid results.


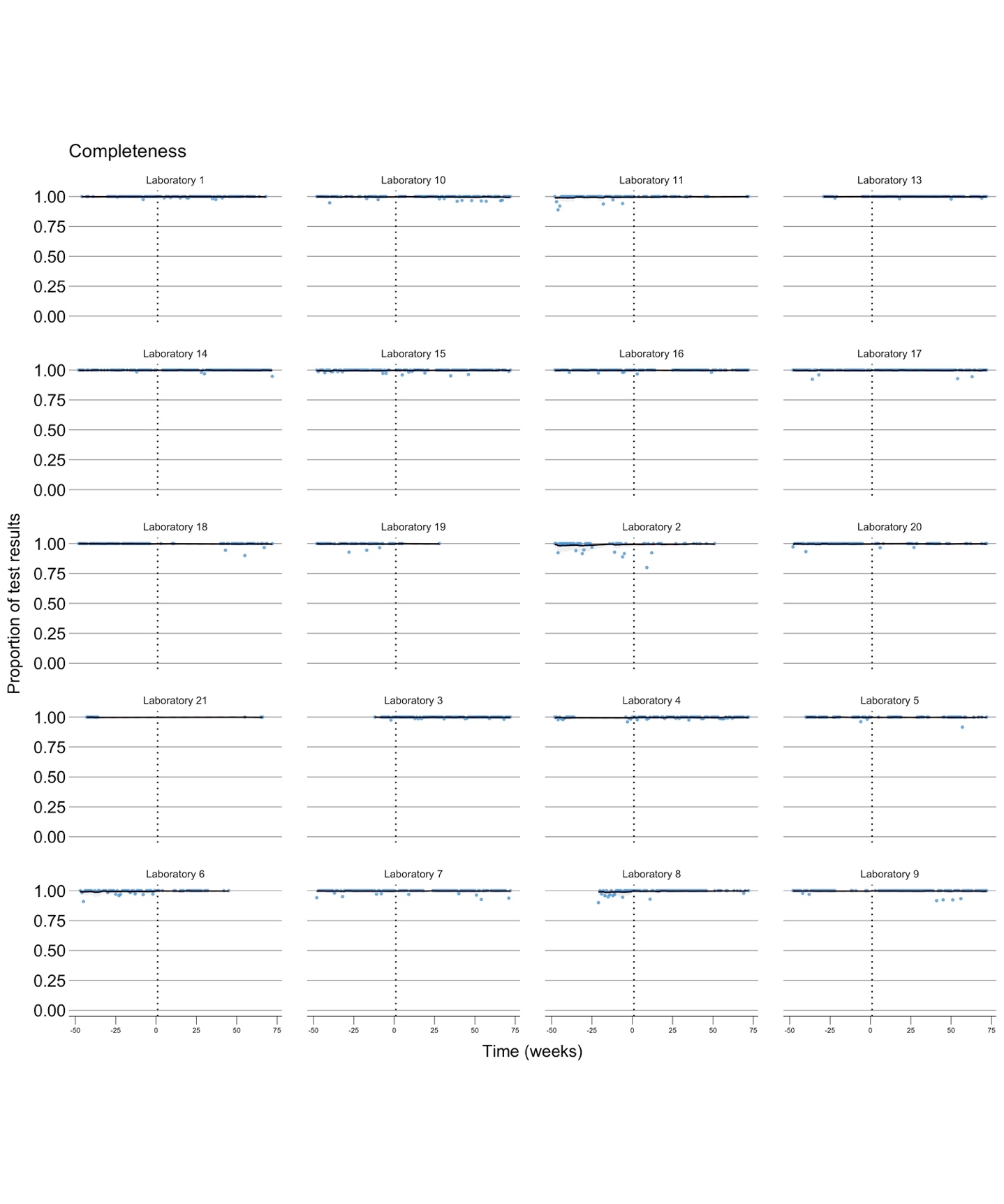


The vertical dotted line represents the first week when a laboratory started using OpenELIS. The blue dots represent the observed outcomes. The black solid line is the estimated trend around the observed data points, representing the factual. The light gray area around the solid black line represents the 95% prediction interval around the factual estimates. The red dashed line represents the counterfactual estimates if there were no OpenELIS implementation.

Regional reference laboratories: Laboratories 1, 2, 3, 9, 13

Regular regional laboratories (non-reference): 10, 11, 12

Laboratories at general hospitals or urban hospitals: Laboratories 4, 5, 6, 7, 8, 14, 15, 16, 17, 18, 19, 20, 21
